# Editorial: Organoids for drug discovery

**DOI:** 10.3389/fphar.2025.1732060

**Published:** 2025-11-19

**Authors:** Chongyang Shen, Mingliang You, Lingzhi Wang

**Affiliations:** 1 Basic Medicine School, Chengdu University of Traditional Chinese Medicine, Chengdu, China; 2 Hangzhou Cancer Hospital, Hangzhou, China; 3 Cancer Science Institute of Singapore, National University of Singapore, Singapore, Singapore

**Keywords:** organoids, iPSCs, drug screening, drug response prediction, drug efficacy and toxicity

The term “Organoid”, coined by Hans Clevers in 2009, refers to structures that closely mimic the architecture and function of specific organs. He focuses on elucidating the role of Wnt signaling in tissue homeostasis and oncogenesis. The discovery of Lgr5 as a marker of Wnt-stem cells in tissue led to the development of a technology that generates epithelial organoids from single stem cells.

Organoids retain key characteristics of the original tissue, making them suitable for studying physiology and pathology of diseases. (Patient derived organoids, PDO), which function as avatars, can also predict the most effective drugs for individual patients. Organoids models represent valuable approaches for studying cancer development and drug resistance, and their *in vitro* culture allows extensive manipulations, such as genetic modification. Researchers have constructed organoids of specific lineages, including brain, liver, kidney, lung, intestinal, and skin, using human embryonic stem cells (hESCs) or induced pluripotent stem cells (iPSCs). Organoids derived from hESCs or iPSCs recapitulate numerous features, such as high structural complexity, size, and organization.

However, most organoids lack the stromal, immune, neural, and vascular endothelial cell, limiting their utility in disease modeling. To model whole-body physiology and systemic diseases, multi-organ interaction chip with recirculating vascular flow and real-time monitoring system are required. Therefore, organoids models incorporating these key components represent an emerging platform with significant potential for evaluation of new drug efficacy and toxicity.

On 10 April 2025, the FDA announced plan to phase out animal testing, replaced using (New Approach Methodologies, NAM), including (Artificial Intelligence, AI) computational models and organoids based efficacy and toxicity testing. Al, particularly deep Learning models, can now design completely novel molecular structures that have never been synthesized and predict the strength of drug-target interactions as well as potential toxicity. The integrating of AI, organoids, and computational biology has emerged as a transformative approach for exploring novel therapeutic strategies and providing human-relevant toxicity data.

This Research Topic aims to translate basic scientific discoveries into clinical applications and highlight new researches in the field of organoids. The included studies cover the major areas of growing interest in organoids research. Shen et al. developed an iPSC derived kidney organoid exhibiting renal tubular and glomerular structures and expressing specific kidney markers. This kidney organoid based prediction system was used to assess the protective effects of celastrol against cisplatin-induced nephrotoxicity. Wang et al. presented the strengths and weaknesses with the utilization of the ECM in kidney organoid culture. To explore the utility of organoid models in elucidating the reproductive complications of neurodrug exposure, Mariam et al. reviewed the principles of organoid models, emphasizing their ability to recapitulate neurodevelopmental processes and simulate drug-induced toxicity in a controlled environment. Xu et al.summarized organoid models developed for studying the mechanisms of diabetes and its complications, as well as for drug screening. Zou et al. introduced the definition and advantages of organoids and described their application in benign and malignant liver and biliary tract diseases, drug research, and regenerative medicine. In recent years, the application of PDO in drug detection and screening has rapidly expanded. Zhou et al. reviewed the application of colorectal organoid technology in basic methods and explores the pathogenesis of and personalized treatment of various colorectal diseases. Shen et al.summarized the use of organoids in modeling, drug efficacy assessment, and drug response prediction for ovarian, endometrial, and cervical cancers, offering valuable options for gynecological oncology patients.

We, the editors from this Research Topic, thank all authors for their contributions. We also extend our sincere gratitude to the editors and reviewers for their dedicated efforts in reviewing, revising, and providing timely feedback on the manuscript.

